# Reliability and Validity of the Alberta Context Tool (ACT) with Professional Nurses: Findings from a Multi-Study Analysis

**DOI:** 10.1371/journal.pone.0127405

**Published:** 2015-06-22

**Authors:** Janet E. Squires, Leslie Hayduk, Alison M. Hutchinson, Ranjeeta Mallick, Peter G. Norton, Greta G. Cummings, Carole A. Estabrooks

**Affiliations:** 1 School of Nursing, University of Ottawa, Ottawa, Ontario, Canada; 2 Ottawa Hospital Research Institute, Ottawa, Ontario, Canada; 3 Department of Sociology, University of Alberta, Edmonton, Alberta, Canada; 4 School of Nursing and Midwifery, Faculty of Health, Deakin University and Cabrini Institute, Melbourne, Australia; 5 Ottawa Methods Centre, Ottawa Hospital Research Institute, Ottawa, Ontario, Canada; 6 Department of Family Medicine, University of Calgary, Calgary, Alberta, Canada; 7 Faculty of Nursing, University of Alberta, Edmonton, Alberta, Canada; Canadian Agency for Drugs and Technologies in Health, CANADA

## Abstract

Although organizational context is central to evidence-based practice, underdeveloped measurement hindersitsassessment. The Alberta Context Tool, comprised of 59 items that tap10 modifiable contextual concepts, was developed to address this gap. The purpose of this study to examine the reliability and validity of scores obtained when the Alberta Context Tool is completed by professional nurses across different healthcare settings. Five separate studies (N = 2361 nurses across different care settings) comprised the study sample. Reliability and validity were assessed. Cronbach’s alpha exceeded 0.70 for9/10 Alberta Context Tool concepts. Item-total correlations exceeded acceptable standards for 56/59items. Confirmatory Factor Analysescoordinated acceptably with the Alberta Context Tool’s proposed latent structure. The mean values for each Alberta Context Tool concept increased from low to high levels of research utilization(as hypothesized) further supporting its validity. This study provides robust evidence forreliability and validity of scores obtained with the Alberta Context Tool when administered to professional nurses.

## Introduction

International awareness and acceptance of the importance of organizational context to evidence-based practice and to better patient outcomes is growing. Little empirical evidence supports these claims, though, in part because we lack a robust measure oforganizational context.Several instruments measure selected aspects of context, for example organizational culture[1,2], organizational climate[3,4], and the practice environment[5,6]. However, these tend to be lengthy (potentially increasing respondent burden) and do not capture a broad conceptualization of context, making them often not feasible for use in the busy, resource-stretched healthcare settings where healthcare providers frequently practice. In 2006, the Alberta Context Tool (ACT) was developed to address this important empirical gap. There are now sufficient data across multiple settings to conduct an advanced psychometric assessment of the instrument’s performance when administered to professional nurses. Confirming adequate measurement of the ACT would allow itto be used with increased confidence.

Organizational context is “…the environment or setting in which people receive healthcare services, or in the context of getting research evidence into practice, the environment or setting in which the proposed change is to be implemented” [7]. According to the *Promoting Action on Research Implementation in Health Services* (PARiHS) framework, research implementation/utilization occurs as a result of the interplay between three core concepts: evidence, context, and facilitation[8]. These authors’ conceptualization of context was based on literature from the fields of quality improvement, organizational excellence, learning organizations, and change management. They proposedthat context is comprised of three concepts (leadership, culture, and evaluation), each of which exists on a continuum from low to high. Expanded views of organizational context can be found in related literature. Glisson[9], for example, includes additional dimensions such as organizational structure (centralization of power and formalization of roles), work attitudes, hard and soft core technologies (raw materials, knowledge, skills, and equipment), and inter-organizational domains (organizations linked by a common societal problem or set of problems).

Strong hypotheses about the central role of context in research use and outcomes have led to large but distinct bodies of literature on context (e.g., [9–12]. Several characteristics of context are identified in this literature as potentially important to the use of research by healthcare providers. Documented in this literature are contextual characteristics that can increase an individual’s use of research evidence. These characteristics include: specialist services and resources; presence of professional standards; positive attitudes and a higher proportion of managers; continuity in management; organizational slack; effective communication and collaboration between departments; presence of opinion leadership; senior management support for evidence-based practices; features related to organizational culture and climate (including leadership style); and, social interaction. Many of these characteristics are potentially modifiable, thus they could be targets of future tailored implementation efforts *if*, through robust measurement, they can be shown to consistently and positively influence research use and/or improve outcomes. The ACT was specifically developed to assess modifiable dimensions of organizational context in relation to care providers’ and managers’ use of research evidence in practice [13–15]. It is also beginning to be investigated in relation to healthcare provider outcomes, e.g., aggression from residents [16].

### The Alberta Context Tool (ACT)

Underpinned by the PARiHS framework[8] and related literature[10,11,17], the ACT is designed to measure modifiable dimensions of organizational context as perceived by individual healthcare professionals. Its development was guided by standard methods of survey design, balanced with a practical requirement for brevity, given that it would be administered to nurses working in resource-constrained environments. The initial version of the instrument was designed for nurses in acute care hospitals and contained 56 items representing 10 core concepts: leadership, culture, evaluation, social capital, formal interactions, informal interactions, structural and electronic resources, organizational slack–staff, organizational slack–space, and organizational slack–time. Table 1 defines these 10 context concepts, lists sample items, and how scored. The ACT has since been adapted for additional provider groups (healthcare aides, physicians, allied health professionals, specialists/educators, and care managers) and settings (adult hospitals, pediatric hospitals, residential long-term care facilities (nursing homes), and community/home care).The instrument has or is currently being used in 56studies (24 studies in adult hospitals, 8 studies in pediatric hospitals, 17 studies in long-term care settings, and 7 studies in home care settings) across 8 countries (Canada, USA, Sweden, Netherlands, United Kingdom, Republic of Ireland, Australia, and China) and is available in six languages (English, French, Swedish, Dutch, German, and Mandarin).

**Table 1:** Alberta Context Tool (ACT) Concepts.

Concept	Definition	Expected relationship to research use	Sample item
Leadership ^1^	The actions of formal leaders in an organization (unit) to influence change and excellence in practice, items generally reflect emotionally intelligent leadership	Care providers who perceive more positive (emotionally intelligent) unit leadership report higher research use	Calmly handles stressful situations
Culture ^1^	The way that “we do things’ in our organizations and work units, items generally reflect a supportive work culture	Care providers who perceive a more positive unit culture report higher research use	My organization effectively balances best practice and productivity
Evaluation ^1^	The process of using data to assess group/team performance and to achieve outcomes in organizations or units (i.e., evaluation)	Care providers who perceive a larger number of unit feedback mechanisms report higher research use	Our team routinely monitors our performance with respect to action plans
Social Capital ^1^	The stock of active connections among people. These connections are of three types: bonding, bridging, and linking	Care providers who perceive more positive unit social capital activities report higher research use	People in the group share information with others in the group
Formal Interactions ^2^	Formal exchanges that occur between individuals working within an organization (unit) through scheduled activities that can promote the transfer of knowledge	Care providers who perceive a larger number of formal unit interactions report higher research use	How often do these activities occur?
			-Team meetings
Informal Interactions ^2^	Informal exchanges that occur between individuals working within an organization (unit) that can promote the transfer of knowledge	Care providers who perceive a larger number of informal unit interactions report higher research use	How often do you interact ….?
			- Someone who *champions* research and its use in practice
Structural/ Electronic Resources ^3^	The structural and electronic elements of an organization (unit) that facilitate the ability to assess and use knowledge	Care providers who perceive a larger number of unit structural and electronic resources report higher research use	How often do you use/attend the following?
			- A Library
Organizational Slack	The cushion of actual or potential resources which allows an organization (unit) to adapt successfully to internal pressures for adjustments or to external pressures for changes		
Staff ^1^		Care providers who perceive sufficient unit staffing levels report higher research use	Enough staff to deliver quality care
Space ^1^		Care providers who perceive having sufficient space on their unit report higher research use	Use of designated space
Time ^1^		Care providers who perceive having sufficient time on their unit report higher research use	Time to do something extra for patients

^1^ = Scale: 1-strongly disagree; 2-disagree; 3-neither agree or disagree; 4-agree; 5-strongly agree

^2^ = Scale: 1-never; 2-rarely; 3-ocasionally; 4-frequently; 5-almost always

^3^ = Scale: 1-never; 2-rarely; 3-ocasionally; 4-frequently; 5-almost always; 6- not accessible

During its initial development, the ACT was assessed for *content* validity (do the items embody the content of its respective concept) and *response processes* validity (respondents ‘understanding and interpretation of the various items)[18].Content validity was estimated by the research team responsible for its development, who are recognized as international experts in the areas of organizational context and research utilization [19,20]. Response processes validity was estimated using focus groups with care providers (nurses, healthcare aides, physicians, allied health professionals, specialists/educators, and care managers)[19,21,22]. In addition to content and response processes validity evidence, two standard reliability and validity investigations of scores obtained using the ACT were conducted[14,20]. The first investigation, conducted with data from a sample of pediatric nurses, reported a principal components analysis showing 13-factors; 2 of the 10 ACT concepts(informal interactions and structural and electronic resources) broke into multiple factors within their proposed concept. Hence, the theory behind the ACT remained at 10 concepts overall. Adequate *internal consistency* reliability was also reported; Cronbach’s alpha coefficients exceeded .70 for 7 of the 10 ACT concepts (exceptions: formal interactions, structural resources, and organizational slack-space) [20]. The second investigation used ACT scores from unregulated care providers (healthcare aides)working in residential long-term care facilities[14]. In this investigation a confirmatory factor analysis (CFA) was conducted; findings revealed that the ACT data collected was consistent with the structure suggested in the ACT instrument. Internal consistency reliability was again adequate; 8ofthe 10 ACT concepts had alpha>0.70 (exceptions: formal interactions and organizational slack-space) [14].

### Psychometric Framework

The *Standards for Educational and Psychological Testing*(the *Standards*) guided the advanced psychometric assessment of the ACT presented in this paper, particularly the assessment of validity. The *Standards* are described as ‘best practice’ in psychometrics [23]. Validity, using this approach, refers to “the degree to which evidence and theory support the interpretations of test scores entailed by proposed uses of tests’ content”[18]. With this approach, validation involves accumulating evidence from four sources: (a) *test-content*–do the items cover the content of the construct being measured; (b) *response processes*–how respondents interpret, process, and elaborate on item content and whether this is in accordance with the construct; (c) *internal structure*–how the items relate to one another; and, (d) *relations to other variables*–relationships between scores obtained with the instrument and other variables which the score is (or is not) expected to relate[18,24]. This later type of validity evidence (*relations to other variables*) can come from correlations, statistical modeling, and group-comparison studies. Previously, various different validity labels were used to refer to this type of validity evidence, for example: criterion-related validity (i.e., concurrent validity, predictive validity), convergent validity, and discriminant validity. By collecting data that tap each of these four validity sources (content, response processes, internal structure, and relations to other variables), we were able to use all available data to assess the comprehensiveness of the validity evidence for scores obtained with the ACT administered to nurses to inform a comprehensive validity argument. In previous work, the ACT was assessed with nurses for preliminary signs of validity, using test content and response processes evidence[19,21,22]. In this study we add to this previous ACT validity evidence by assessing specifically the scores obtained from its use with nurses for advanced sources of validity evidence: internal structure and relations with other variables.

### Purpose

The purpose of this study was to examine the reliability and validity of scores obtained when the ACT is completed by professional nurses across different healthcare settings. Our specific study aims were: (1) to assess the internal consistency of the 10 ACT concepts when completed by nurses; (2) to assess the internal structure and relations to other variables validity of the 10 ACT concepts when completed by nurses.

## Methods

### Design and Participants

This study was a secondary analysis of data from five separate studies (Table 2),each employing a cross-sectional descriptive survey design. Data included in our analyses are from professional (i.e., registered/licensed) nurses who completed the ACT, questions on demographic variables, and the Estabrooks measure of either instrumental and/or conceptual research utilization [25]. The nurses were from two countries (Canada and Australia) and various clinical settings (long-term care, acute pediatric hospitals, acute adult hospitals, and community/home care).

**Table 2:** Data Collections Analyzed from Five Studies Utilizing the ACT.

Data File	Study Name	Study Purpose	Care Setting	Country	Nurse Sample Size	Data Collection Period
1	Translating Research in Elder Care (TREC) (Project 1)	To examine the influence of context on research utilization and the subsequent impact of research utilization on healthcare provider outcomes in 36 nursing homes in Western Canada	Residential Long Term Care (i.e., Nursing Homes)	Canada	325	06/2008-07/2009
2	The CIHR Team Grant in Children’s Pain (Project 2)	To examine the influence of context on research utilization and provider outcomes in eight pediatric hospitals across Canada	Acute Pediatric Hospitals	Canada	819	05/2011-06/2011
3	The Role of PDAs in Evidence-Based Practice	To evaluate organizational context and individual variables that influence nurses’ use of personal digital assistants (PDAs) or tablet personal computers to access information resources to support clinical decision making and patient care.	Residential Long Term Care (i.e., Nursing Homes)	Canada	702	04/2009-03/2010
			Acute Pediatric Hospitals			
			Acute Adult Hospitals			
			Community/ Home Care			
4	Linking Best Practice Guideline (BPG) Use and Health Outcomes for Better Information and Care in the Community (HOBIC)	To explain throughput processes such as BPG knowledge utilization and its impact on HOBIC nurse sensitive outcomes	Community/ Home Care	Canada	348	04/2009-03/2010
5	The Older Person and Improving Care (TOPIC 7)	To examine seven teams of nurses in their efforts to improve one basic aspect of care for older people in adult hospitals.	Acute Adult Hospitals	Australia	224	09/2008-12/2008

### Instrument

#### The ACT

The ACT was developed to elicit individual care providers’ perceptions of their organizational context. Three principles informed its development: (a) the PARiHS framework and related literature;(b) brevity–an instrument that could be completed in 20 minutes (nurses required an average of 9.1 minutes to complete the ACT online and 13.7 minutes on paper);and (c) a focus on modifiable contextual concepts [20]. The first version of ACT(nurses, adult hospitals) was developed between May 2005 and September 2006, in four phases: comprehensive literature review, conceptual refinement, item construction, and feasibility assessment. Because item wording, particularly in the stem statements, required modification for different provider groups, versions of the ACT specific to each of five professional subgroups (nurses, physicians, allied health professionals, specialists, and managers) were developed. The ACT was subsequently modified for all five professional subgroups in pediatric hospitals, residential long-term care facilities (nursing homes) and community/home care settings. An additional version was developed for healthcare aides in nursing homes [20].

The ACT Nurse Version reported in this paper contains 56–59 items (depending on the care setting: acute care has 56 items while homecare has 57 items and long-term care has 59 items). The difference in the number of items reflects setting specific contexts; the homecare version has an item on access to a computer that is not required in acute care version, and the long-term care version has this item plus an additional two items on (1) interactions with care aides and (2) having enough staff to ensure residents have their best day. All three ACT Nurse Versions assess 10 core contextual concepts: leadership, culture, evaluation, social capital, formal interactions, informal interactions, structural and electronic resources, organizational slack–staff, organizational slack–space, and organizational slack–time (see Table 1 for definitions, sample items, and scoring). While all items are asked using Likert or frequency rating scales, the items for three of the concepts (formal interactions, informal interactions, structural and electronic resources) were not designed as true scales. The later item sets represent, for example, a list of people one may (or may not) interact with or a list resources one may (or may not) have access to, rather than a cohesive set of item tapping a shared concept. These item sets are therefore recoded as existing or not and a sum is then taken to derive an overall score for the concept. These item sets are referred to as non-scaled items in this paper. The remaining ACT concepts were designed as true scales; the mean of all items in each concept’s item set was used to derive an overall score for that concept. These item sets are referred to as scaled items in this paper

#### Research Utilization

Organizational context is theorized to be important to research utilization by care providers [8]. In this study, in addition to measuring context with the ACT we measured two kinds of research utilization: *instrumental* and *conceptual*. Instrumental research utilization (IRU) is research use that results in an observable action (e.g., use of a best practice guideline [26]. Conceptual research utilization (CRU), on the other hand, is the cognitive research use which may or may not lead to observable actions [26]. Both IRU and CRU were measured with a single question asking respondents how often they use research in the described way and was scored on a five-point scale from 1 (use less than 10% of the time) to 5 (use almost 100% of the time).

### Data Collection

We analyzed five studies utilizing the ACT that collected data from professional nurses between June 2008 and June 2011. Prior to our psychometric analyses of the data, we reconfigured those five datasets; this entailed detailed mapping of the data files to link the individual scale instructions, items and response options across the individual datasets to create a single (master) nurse dataset. Our research team decided jointly which items could and could not be combined in merging the five datasets into that single dataset.

### Data Analysis

Data were analyzed using Statistical Analysis Software (SAS Institute) and LISREL (Scientific Software International, Inc) statistical software packages. No items had significant missing data (i.e., all items were answered by 90% or greater of respondents) [27]. Sample demographic characteristics were summarized using descriptive statistics. All analyses were carried out for each individual healthcare setting (findings not reported); similar results were found across settings, therefore the analyses reported in this paper reflect all settings combined.

#### Reliability

Reliability for each ACT concept (n = 10) was assessed using Cronbach’s alpha; while an alpha of 0.70 is thought of as acceptable, 0.80 or higher is preferred for established scales such as those contained within the ACT [28–30].

#### Validity

Our validity testing included an assessment of the *internal structure* of the ACT as well as an assessment of *relations with other variables* between the ACT scores and research utilization, which according to the PARiHS framework, should be related.

#### Validity: Internal structure validity evidence

We conducted item-total statistics (in SAS)andConfirmatory Factor Analysis (CFA)(in LISREL) in this phase. Item-total statistics were calculated for the items in each of the 10 ACT concepts; we considered items for reassessment if (a) they correlated with their scale score at 0.3 or lower, and (b) they caused a substantial rise or fall in the Cronbach’s alphavalues that were observed when we recalculated alpha on a reduced set of items (i.e., without the item) [28,31]. We used CFA to confirm the latent structure of the ACT that was observed in our earlier work with healthcare aides[14].In developing the ACT, items selected to measure each of the 10 concepts were written to assess similar but explicitly non-redundant elements of the concept. To fully comply with CFA structuring, items within each concept would have to be entirely redundant (except for measurement error)[32]. For proper factor model specification, the errors should be ‘independent’, and the entire coordination of the items within sets should depend exclusively on the relevant latent factor–which is another way of saying the items are redundant because they are similar to one another only to the extent of their common dependence on a latent factor and differ from one another in only error-ways (where those errors are to be minimized and made as independent or random as possible). Thus, our intentional differentiation of items within each concept implies that the CFA model is not absolutely precise, even though the strong differentiation between the 10 concepts makes the CFA model the most appropriate of the available models for assessing internal consistency.

We examined three factor models, informed by previous ACT work with healthcare aides [14]. Model 1 included all ACT items, Model 2 included the items contained in the seven scaled ACT concepts and Model 3 included the three non-scaled ACT concepts. We tested model–data fit with χ^2^, to determine the consistency between the model-implied covariance matrix (from CFA) and the sample covariance matrix (from our data); a significant χ^2^ value implies detectable ill fit. We also report common ‘close-fit’ indices: (1) the root mean square of approximation (RMSEA); (2) the standardized root mean square residual (SRMSR); and, (3) the comparative fit index (CFI). A RMSEA < 0.06 and SRMSR < 0.09 [33,34] and a CFI value > 0.90 [33,35] indicate 'close fit'.We anticipated that our CFA models would be able to detect some ill fit due to our deliberate use of non-redundant items. Substantial loadings within factors constitute the most compelling evidence from the CFA analysis, given the non-redundancy of items between the 10 ACT scales.

#### Validity: Relations to other variables validity evidence

We calculated Pearson’s correlation coefficients between the 10 ACT concepts and IRU and CRU. Cohen’s [36] criteria were used to describe the magnitude of the correlations as small (r = 0.10), moderate (r = .30), or strong (r = .50 or higher). Following calculation of Pearson’s correlation coefficients, we examined each ACT score to see if its mean score changed(and in what direction) as scores on IRU and CRU increased; ANOVA was used to determine if the changes in mean scores were statistically significant.

### Ethical Considerations

The University of Alberta Research Ethics Board approved this study (Pro00016573). Because this study is a secondary analysis of existing anonymous survey data, informed consent from the participants of the original studies neither was possible nor considered necessary by the research ethics board above. Consent in the original studies was through submission of an anonymous online survey. No clinical records or patient data were collected in this study or in the original studies. The data collected and used in this study was anonymized survey data from healthcare professionals on their perceptions of their work environment (context) and their self-reported daily use of research evidence.

## Results

### Sample Characteristics

Our sample includes responses from 2361 professional nurses (demographic characteristics in Table 3). Missing data were minimal, with > 90% complete data in all cases (Table 3). ACT concept scores were derived using all available data with missing values treated as missing. The proportion of nurses across the different healthcare settings was: adult hospitals (27%), pediatric hospitals (35%), long term care (18%), and community/home care (20%).

**Table 3:** Characteristics of Study Sample (N = 2361).

Demographic Characteristics		N (%)
Gender	Female	2167 (91.78)
	Male	153 (6.48)
	*Missing*	*41 (1*.*74)*
Age	20–29 years	581 (24.61)
	30–39 years	567(24.02)
	40–49 years	570 (24.14)
	50–59 years	490 (20.75)
	60–70 years	136 (5.76)
	>70 years	2 (0.08)
	*Missing*	*15 (0*.*64)*
Highest Education	Diploma/Certificate	1228 (52.01)
	Bachelors Degree	1002 (42.44)
	Masters Degree	89 (3.77)
	PhD Degree	3 (0.13)
	Medical Degree	2 (0.08)
	*Missing*	*37 (1*.*57)*
Setting	Adult Hospitals	637 (26.98)
	Pediatric Hospitals	819 (34.69)
	Long Term Care	434 (18.38)
	Community/Home Care	471 (19.95)
Years Worked in Current Position [Mean (SD)]		9.60 (9.43)
Years worked on Unit/Facility [Mean (SD)]		7.58 (7.62)

### Reliability

Reliability coefficients for the ACT concepts are documented in Table 4. Nine of the ten ACT concepts had alpha coefficients that were at or exceeded the accepted standard of 0.80 for established scales [28–30]; the only exception was ‘formal interactions’ which had an alpha of 0.59.

**Table 4:** Item Characteristics by ACT Concept (N = 2361).

ACT Concept	No. Items	Score Range	No. Completed Responses	Mean Response	Standard Deviation	Reliability		
						Item-Total Correlation^1^ (Range)	Cronbach Alpha	Alpha after item deleted
**Leadership**	6	1–5	2307	3.748	0.793	(0.681, 0.815)	0.91	(0.88, 0.90)
**Culture**	6	1–5	2298	3.894	0.578	(0.520, 0.573)	0.80	(0.75, 0.77)
**Evaluation**	6	1–5	2299	3.202	0.861	(0.717, 0.841)	0.92	(0.90, 0.92)
**Social Capital**	6	1–5	2315	3.969	0.518	(0.509, 0.622)	0.80	(0.76, 0.78)
**Formal Interactions**	4	0–4	2286	1.444	1.087	(0.134, 0.492)	0.59	(0.42, 0.69)
**Informal Interactions**	9	0–9	2253	4.284	2.042	(0.368, 0.640)	0.80	(0.76, 0.79)
**Structural and Electronic Resources**	11	0–11	1288	3.866	2.530	(0.281, 0.611)	0.80	(0.77, 0.80)
**Organizational Slack:**								
*Time*	4	1–5	2320	2.967	0.645	(0.576, 0.654)	0.80	(0.72, 0.76)
*Space*	3	1–5	1611	2.714	1.072	(0.466, 0.813)	0.80	(0.53, 0.88)
*Staff*	2	1–5	2334	3.066	1.069	(0.758)	0.86	N/A

An extra item is asked in the long term care (LTC) version (Informal Interactions–interactions with healthcare aides; Staff—We have enough staff to make sure residents have the best day.

^1^ Item-Total Correlation = correlation between each item in an item set and the overall score (e.g., mean) for that item set; values > 0.30 are considered acceptable

### Validity

#### Internal structure: Item-total correlations and statistics

Almost all (56/59, 95%) of the ACT items had corrected item-total correlations greater than the predetermined cut-off of 0.3 (Table 4). The three items that did not meet this minimum cut-off are from three different ACT concepts: (a) formal interactions (item *Continuing education outside nursing home*, 0.134); (b) informal interactions (LTC version; item *Hallway talk*, 0.252); and (c) structural and electronic resources (item *Library use*, 0.281). No substantial rises or falls were found between the original (all items in the subscale for the concept) and the recalculated (i.e., individually with each item removed) Cronbach’s alpha values for each ACT concept, giving further evidence of internal structure validity.

#### Internal structure: Confirmatory factor analysis

We estimated three factor models. Model 1 contained all ACT items i.e., a 10-factor model in which each ACT item loaded on only its corresponding ACT concept. Model 2 examined the seven ACT *scaled* concepts, and Model 3 examined the three ACT *non-scaled* concepts (Table 5).Overall, Model 2 had the best fit, followed by Model 1 and then Model 3. Correlations between the 10 concepts are mostly in the moderate to large range in magnitude according to Cohen’s standards (Table 6).The fit indices of the full 10-factor model (Model 1) and the seven-factor model (Model 2) would be interpreted as ‘close fit’ using conventional standards (RMSEA, SRMSR, CFI) but, as anticipated; the χ^2^ test does not support the *precise* fit of any of the models (Table 5).We anticipated that Model 2 would provide the best fit because it contained only scaled items; items in the three non-scaled concepts (included in Models 1 and 3) were developed to reflect elements that are less dependent on a common cause than are those within the scaled concepts. Therefore, as expected, the χ^2^ and close fit indices are noticeably superior for Model 2 compared to Models 1 and 3 (Table 5).

**Table 5:** Completely Standardized Factor Loadings and Model–Data Fit (N = 2361).

Concept	Item^1^	Factor Loadings		
		Model 1	Model 2	Model 3
**Leadership**	Looks for feedback	0.751	0.751	
	Focuses on successes	0.713	0.712	
	Calmly handles stress	0.782	0.782	
	Listens, acknowledges, responds	0.863	0.863	
	Actively mentors and coaches	0.819	0.819	
	Resolves conflicts	0.822	0.822	
**Culture**	Receive recognition	0.584	0.584	
	Supportive work group	0.630	0.630	
	Organization balances	0.670	0.670	
	Professional development	0.670	0.671	
	Clear on what patients want	0.606	0.606	
	Control over work	0.590	0.591	
**Evaluation**	Routinely receive information	0.743	0.741	
	Discusses data informally	0.783	0.783	
	Formal process	0.808	0.808	
	Formulates action plans	0.901	0.902	
	Monitors our performance	0.899	0.899	
	Compares our performance	0.748	0.747	
**Social Capital**	Share information with others	0.635	0.631	
	Group participation is valued	0.717	0.720	
	Information is shared	0.598	0.589	
	Aim is to help others	0.561	0.571	
	Observations are taken seriously	0.678	0.677	
	Comfortable talking in authority	0.616	0.616	
**Slack—Staff**	Get the *necessary w*ork done	0.805	0.805	
	Deliver best possible care	0.942	0.942	
**Slack—Space**	Adequate space	0.514	0.508	
	Private space	1.022	1.033	
	Use of private space	0.821	0.813	
**Slack—Time**	Do something extra for patients	0.661	0.666	
	Talk about plan of care	0.680	0.672	
	Look something up	0.707	0.705	
	Talk about new clinical knowledge	0.763	0.768	
**Informal Interactions**	Colleagues in my identical field	0.575		0.566
	Physicians	0.731		0.728
	Other healthcare providers	0.770		0.768
	Research nurse or coordinator	0.530		0.538
	Clinical educator/instructor	0.715		0.720
	QI representative	0.571		0.579
	Champion	0.546		0.551
	‘Hallway talk’	0.435		0.423
	Informal bedside teaching	0.528		0.524
**Formal Interactions**	Team meetings	0.646		0.639
	Patient rounds	0.736		0.741
	Family conferences	0.673		0.679
	Continuing education	0.198		0.186
**Structural/ Electronic Resources**	Library	0.350		0.344
	Textbooks	0.422		0.420
	Journals	0.559		0.549
	Notice boards	0.600		0.609
	Policies and procedures	0.783		0.797
	Clinical practice guidelines	0.760		0.772
	In-services	0.429		0.421
	Computer hooked to internet	0.489		0.472
	Computerized decision support	0.368		0.356
	Reminders	0.421		0.406
	Websites	0.447		0.432
**Model-Data Fit:**				
**χ** ^**2**^ **(*p-*value)**		13,469 (< .001)	2,783 (< .001)	7,598 (< .001)
		df = 1494	df = 474	df = 249
**RMSEA** ^**2**^		0.0671	0.0466	0.130
**SRMR** ^**3**^		0.0563	0.0417	0.0884
**CFI** ^**4**^		0.935	0.977	0.855

^1^ The horizontal lines separate factors within each model (i.e., there are10 factors in Model 1, seven in model 2 and three in Model 3)

^2^RMSEA = root mean square error of approximation

^3^SRMR = standardized root mean squared residual

^4^CFI = Comparative Fit Index

**Table 6:** Correlations between the 10 ACT Latent Concepts (N = 2361).

	Leadership	Culture	Evaluation	Social Capital	Formal Interactions	Informal Interactions	Structural/ Electronic Resources	Org Slack–Staff	Org Slack–Space	Org Slack–Time
**Leadership**	1.000									
**Culture**	0.611	1.000								
**Evaluation**	0.409	0.534	1.000							
**Social Capital**	0.115	0.235	0.201	1.000						
**Formal Interactions**	0.393	0.608	0. 367	0.390	1.000					
**Informal Interactions**	0.400	0.628	0.465	0.185	0.510	1.000				
**Structural/ Electronic Resources**	0.225	0.347	0.438	0.777	0.440	0.289	1.000			
**Org Slack–Staff**	0.166	0.265	0.346	0.663	0.353	0.257	0.886	1.000		
**Org Slack–Space**	0.136	0.221	0.282	0.544	0.249	0.148	0.664	1.00	1.000	
**Org Slack–Time**	0.082	0.149	0.186	0.332	0.199	0.188	0.320	0.447	0.369	1.000

Factor loadings for all three models were in the predicted direction. The magnitude of the loadings was moderate to high for the scaled concepts (Model 1, 2). The loadings for the non-scaled items tended to be smaller regardless of whether these items appeared alone (Model 3) or accompanying the scaled items (Model 1). The loadings for nine ACT concepts are sufficiently large and uniform to justify clustering the items within those contextual concepts. However, items in structural and electronic resources appear have more disparate causes, rather than sharing a single underlying cause.

#### Relations to other variables: Correlations and increasing mean value analysis

Correlation among the 10 latent factors corresponding to the ACT conceptswith IRU and CRU are presented in Tables 7 and 8 respectively. As expected on the basis of theory, the 10 ACT concepts correlate positively with both IRU and CRU. The magnitude of the associations between the ACT concepts and research utilization were small and similar across instrumental and conceptual research utilization. Although the CFA indicates that the items constituting the structural and electronic resources concept might arise from disparate causes, those items collectively display some of the stronger correlations with both IRU and CRU. We also expected that the ACT concepts would have increasing mean values from lowest to highest levels of IRU and CRU (in line with research utilization theories). Our results support this assumption and thus add to our validity argument (Tables 7 and 8).

**Table 7:** Correlation and Increasing Mean Values of ACT Concepts by Five Levels of Instrumental Research Utilization (IRU) (n = 2094).

	Correlation with IRU^1^	Mean Value (95% Confidence interval (Relative % Change^2^) of ACT Concepts by Level of Instrumental Research Utilization						*p*-value for Mean Difference^3^
		1	2	3	4	5	Total^4^	
Leadership	0.098**	3.64(3.55,3.73)	3.69(3.60,3.78)	3.68(3.60,3.75)	3.82(3.77,3.88)	3.82(3.74,3.90)	3.75(3.72,3.78)	0.004
		(-2.9)	(-1.6)	(-1.9)	(1.9)	(1.9)		
Culture	0.143**	3.79(3.72,3.86)	3.84(3.77,3.91)	3.83(3.77,3.89)	3.94(3.90,3.98)	4.03(3.97,4.08)	3.89(3.87,3.91)	< .0001
		(-2.6)	(-1.3)	(-1.5)	(1.3)	(3.6)		
Evaluation	0.132**	3.02(3.04,3.22)	3.13(3.04,3.22)	3.16(3.08,3.24)	3.28(3.22,3.35)	3.35(3.26,3.44)	3.20(3.17,3.24)	< .0001
		(-5.6)	(-2.2)	(-1.3)	(2.5)	(4.7)		
Social Capital	0.137**	3.90(3.84,3.95)	3.87(3.81,3.94)	3.92(3.87,3.97)	4.02(3.98,4.06)	4.07(4.02,4.12)	3.97(3.95,3.99)	< .0001
		(-1.8)	(-2.5)	(-1.3)	(1.3)	(2.5)		
Formal Interactions	0.192**	0.99(0.89,1.10)	1.30(1.19,1.42)	1.41(1.31,1.51)	1.59(1.50,1.67)	1.60(1.49,1.71)	1.43(1.38,1.47)	< .0001
		(-30.8)	(-9.1)	(-1.4)	(11.2)	(11.9)		
Informal Interactions	0.262**	3.26(3.06,3.47)	3.77(3.55,4.00)	4.17(3.98,4.36)	4.65(4.50,4.80)	4.81(4.62,5.00)	4.25(4.16,4.33)	< .0001
		(-23.3)	(-11.3)	(-1.9)	(9.4)	(13.2)		
Structural/ Electronic Resources	0.185**	3.18(2.95,3.40)	4.01(3.76,4.26)	4.16(3.94,4.39)	4.39(4.22,4.57)	4.55(4.33,4.77)	4.05(3.96,4.14)	< .0001
		(-21.5)	(-1.0)	(2.7)	(8.4)	(12.3)		
Org Slack–Staff	0.093**	2.90(2.78,3.02)	2.98(2.86,3.10)	3.05(2.95,3.15)	3.19(3.11,3.27)	3.14(3.03,3.26)	3.07(3.02,3.11)	0.0004
		(-5.5)	(-2.9)	(-0.7)	(3.9)	(2.3)		
Org Slack–Space	0.114**	3.00(2.90,3.11)	2.96(2.84,3.08)	3.04(2.93,3.14)	3.21(3.13,3.29)	3.28(3.18,3.39)	3.05(3.00,3.09)	< .0001
		(-1.6)	(-3.0)	(-0.3)	(5.2)	(7.5)		
Org Slack–Time	0.151**	2.78(2.71,2.85)	2.93(2.86,3.00)	2.97(2.91,3.03)	3.04(2.99,3.09)	3.08(3.01,3.15)	2.97(2.94,3.00)	< .0001
		(-6.4)	(-1.3)	(0)	(2.4)	(3.7)		

1 = Pearson’s correlation coefficients.

2 = % of difference with respect to the total sample average.

3 = *p*-value for one-way ANOVA using five IRU values.

4 Total = overall mean of the row concept.

** = Significance at 0.01 level

**Table 8:** Correlation and Increasing Mean Values of ACT Concepts by Five Levels of Conceptual Research Utilization (CRU) (n = 1084).

	Correlation with CRU^1^	Mean Value (95%) Confidence interval						*p*-value for Mean Difference^3^
		(Relative % Change^2^)						
		of ACT Concepts by Level of Conceptual Research Utilization						
		1	2	3	4	5	Total^4^	
Leadership	0.128**	3.58(3.44,3.73)	3.60(3.47,3.74)	3.66(3.55,3.76)	3.80(3.72,3.87)	3.83(3.73,3.94)	3.75(3.72,3.78)	0.0025
		(-4.5)	(-4.0)	(-2.4)	(1.3)	(2.1)		
Culture	0.154**	3.75(3.64,3.87)	3.74(3.64,3.83)	3.82(3.75,3.89)	3.88(3.83,3.94)	3.98(3.91,4.06)	3.89(3.87,3.91)	< .0001
		(-3.6)	(-3.9)	(-1.8)	(-0.3)	(2.3)		
Evaluation	0.165**	2.99(2.84,3.15)	3.05(2.92,3.18)	3.24(3.13,3.35)	3.27(3.19,3.35)	3.43(3.31,3.54)	3.20(3.17,3.24)	< .0001
		(-6.6)	(-4.7)	(1.3)	(2.2)	(7.2)		
Social Capital	0.141**	3.83(3.72,3.94)	3.85(3.78,3.92)	3.94(3.88,4.00)	4.02(3.97,4.06)	4.04(3.97,4.11)	3.97(3.95,3.99)	< .0001
		(-3.5)	(-3.0)	(-0.8)	(1.3)	(1.8)		
Formal Interactions	0.184**	1.22(1.04,1.41)	1.50(1.33,1.68)	1.72(1.59,1.86)	1.84(1.74,1.95)	1.93(1.79,2.06)	1.43(1.38,1.47)	< .0001
		(-14.7)	(4.9)	(20.3)	(28.7)	(35.0)		
Informal Interactions	0.249**	3.64(3.27,4.02)	4.42(4.13,4.71)	4.76(4.53,4.99)	5.24(5.06,5.42)	5.30(5.08,5.53)	4.25(4.16,4.33)	< .0001
		(-14.4)	(4.0)	(12)	(23.3)	(24.7)		
Structural/ Electronic Resources	0.224**	3.47(3.08,3.85)	3.86(3.55,4.17)	4.50(4.24,4.75)	4.68(4.48,4.88)	4.97(4.72,5.22)	4.05(3.96,4.14)	< .0001
		(-14.3)	(-4.7)	(11.1)	(15.6)	(22.7)		
Org Slack–Staff	0.114**	2.63(2.43,2.84)	2.98(2.83,3.13)	2.99(2.86,3.12)	3.07(2.96,3.17)	3.14(3.00,3.28)	3.07(3.02,3.11)	0.0006
		(-14.3)	(-2.9)	(-2.6)	(0)	(2.3)		
Org Slack–Space	0.082**	2.98(2.76,3.20)	2.87(2.71,3.04)	2.96(2.81,3.10)	3.05(2.92,3.16)	3.19(3.05,3.33)	3.05(3.00,3.09)	0.0583
		(-2.3)	(-5.9)	(-3.0)	(0)	(4.6)		
Org Slack–Time	0.152**	2.75(2.64,2.87)	2.88(2.79,2.97)	2.91(2.83,2.99)	3.01(2.95,3.07)	3.08(2.99,3.16)	2.97(2.94,3.00)	< .0001
		(-7.4)	(-3.0)	(-2.0)	(1.3)	(3.7)		

1 = Pearson’s correlation coefficients.

2 = % of difference with respect to the total sample average.

3 = *p*-value for one-way ANOVA using five CRU values.

4 Total = overall mean of the row concept.

** = Significance at 0.01 level

## Discussion

This study is the first large-scale psychometric assessment of scores obtained with the Alberta Context Tool (ACT) administered to nurses across multiple healthcare settings. We were guided by the *Standards for Educational and Psychological Testing* (the *Standards*) psychometric framework, which entailed building a solid validity argument based on data addressing four evidentiary perspectives: (a) test content; (b) response processes; (c) internal structure; and (d) relations to other variables [18]. Previous work with nurses estimated test-content and response processes validity and led to refinement of the ACT structure, primarily removal of some items and a reorganization of remaining items under concepts[20]. The ACT was then re-administered to nurses across multiple care settings by multiple research teams (Table 2). In this study, the ACT was tested for additional psychometric properties to complete its validity argument when administered to professional nurses. Our findings support the assertion that the ACT, when administered to nurses, provides a reliable and valid assessment of organizational context.

### Reliability

In developing the ACT, items selected to measure each of the 10 concepts were designed to tap similar yet explicitly non-redundant features. This intentional non-redundancy of the items renders the usual alpha criterion marginally-inappropriate. The items are supposed to be similar within sets (which makes alpha style information somewhat relevant) but the items are not created to be strictly redundant (which makes traditional alpha criteria unlikely to be fully satisfied). Internal consistency reliability of the ACT (Cronbach's alpha coefficients)was at or above the standard (0.80) for established scales administered at the individual level for 9 of 10 concepts. One concept was below this standard: formal interactions (alpha = 0.59). This is consistent with previous assessments with pediatric nurses and healthcare aides in nursing homes [14,20]. The low alpha results partially from the four items within this concept, which were purposefully selected to be non-redundant. Other ACT concepts designed in this manner include informal interactions and structural/electronic resources; the fact that these concepts have acceptable alpha levels may be explained by their larger item sets.

### Validity

#### Internal structure

The items of the ACT were intentionally selected during instrument development to (a) cluster within 10 basic conceptual domains and (b) be non-redundant within each conceptual domain. In initial psychometric assessments of the ACT with nurses, exploratory factor analysis helped to assess and refine the instrument structure[20].In the present study, this refined structure was examined for (a) associations between the items within each ACT concept and (b) evidence of single dimensionality of the seven scaled concepts. The item-total statistics support the refined structure of the ACT, indicating the items within the ACT are linked to their respective concept (Table 4). The item statistics, as expected, also support single dimensionality of the seven scaled concepts.

The intentional clustering of items within the ACT concepts made CFA and factor models an appropriate choice to assess the structure of the ACT. At the same time, because the ACT was designed to include non-redundant items within its concepts, we knew the factor models would not show successful fit. As a consequence of the basic clustering of items into the 10 conceptual domains, but with purposeful non-redundancy of items within the conceptual domains, we anticipated and found high loadings within latent factors but overall significant ill fit of the model. Data from both internal structure assessments (item statistics and CFA) support the structure of the ACT, adding to our validity argument.

#### Relations to other variables

Rounding out our validity argument is *relations to other variables* evidence. The ACT is underpinned by the PARiHS framework[8], which argues that a positive context is important for successful implementation of research into practice (i.e., research utilization) to occur. We expected and found significant correlations between the ACT concepts and IRU and CRU; higher levels of research utilization (irrespective of the kind of research utilization) were significantly associated with more positive contextual conditions as perceived by nurses. Further analyses showed an increase in the mean scores of each of the 10 ACT concepts with increasing mean values from low to high levels of instrumental and conceptual research utilization. These findings are consistent with the assertions in the PARiHS framework and with our psychometric assessment of the ACT administered to healthcare aides [14], again supporting our validity argument for the ACT.

### Limitations and Implications

This study used data from a large sample of nurses in multiple healthcare settings across Canada and Australia. However, to use the ACT with confidence internationally requires testing for psychometric properties of translated versions of the ACT; additional assessments are planned or underway for German, Swedish, and French Canadian versions of the ACT with nurses. This study is also limited by the factor models used to assess *internal structure* validity. While these models were the most appropriate of the available styles of model, a more rigorous test of the theory that underpins the ACT. This however would require additional measures of evidence and facilitation (as proposed in the PARiHS Framework) not available at this time. Therefore, a CFA was the best model choice in the current study. Future assessments of the ACT should include data on evidence and facilitation, in addition to ACT data, to allow a more complete assessment of the theory that underpins the ACT.Other models (e.g., structural equation models), potentially using even single indicators, could also explore the causal structures coordinating the items clustered within the ACT dimensions. When the value of a common (in our model, latent factor) cause changes, all the effected indicators should respond, and the consistency in the items’ responses means the values/scores on the items become correlated or coordinated[32]. We first however need to determine what the ‘best’ indicators are for each of the ACT multi-indicator concepts.

The majority of implications arising from this study and analysis by its nature of being a measurement study focused on reliability and validity relate to future research (as identified above). However, there are also implications for nursing practice. Concepts in the ACT were purposefully selected for inclusion in the tool because they are potentially modifiable. Thus, a measurably reliable and valid ACT holds potential to identify targets for future tailored implementation efforts *if*, through robust measurement, they can be shown to consistently and positively influence research use and/or improve outcomes.

## Conclusion

This study is the first large large-scale psychometric assessment of ACT scores from nurses in a variety of care settings. The results support using the ACT with professional nurses to obtain reliable and valid estimates of organizational context. When combined with the previous preliminary assessment of the ACT with nurses [20], a robust validity argument is formed the provides evidence from all four possible sources of validity presented in the *Standards for Educational and Psychological Testing*, considered ‘best practice’ in psychometrics. We continue to encourage detailed investigation of the items within the ACT concepts whenever the research context permits.
